# Personalized Federated Learning with Hierarchical Two-Branch Aggregation for Few-Shot Scenarios

**DOI:** 10.3390/s26031037

**Published:** 2026-02-05

**Authors:** Yifan Miao, Weishan Zhang, Yuhan Wang, Yuru Liu, Zhen Zhang, Lingzhao Meng, Baoyu Zhang

**Affiliations:** 1 College of Computer Science and Technology, China University of Petroleum (East China), Qingdao 266580, China; 2 Shandong Key Laboratory of Intelligent Oil & Gas Industrial Software, China University of Petroleum (East China), Qingdao 266580, China

**Keywords:** personalized federated learning, few-shot learning, hypernetwork, brain-inspired learning, relation network

## Abstract

Personalized federated learning (pFL) aims to address data heterogeneity by training client-specific models. However, it faces two critical challenges under few-shot conditions. First, existing methods often overlook the hierarchical structure of neural representations, limiting their ability to balance generalization and personalization. Second, recent approaches incorporate representation-level inductive biases that typically rely on rigid assumptions, such as fixed perturbation patterns or compact class clusters, making them vulnerable to distribution shifts in federated environments. To overcome these limitations, we propose pFedH2A, a novel hierarchical framework incorporating brain-inspired mechanisms, tailored for personalized federated learning in few-shot scenarios. First, we design a dual-branch hypernetwork (DHN) that employs two structurally distinct branches to generate aggregation weights. Each branch is biased toward capturing either low-level shared features or high-level personalized representations, enabling fine-grained personalization by mimicking the brain’s division of perceptual and representational processing. Second, we introduce a relation-aware module that learns an adaptive similarity function for each client, supporting few-shot classification by measuring whether a pair of samples belongs to the same class without relying on rigid prototype assumptions. Extensive experiments on public image classification datasets demonstrate that pFedH2A outperforms existing pFL baselines under few-shot scenarios, validating its effectiveness.

## 1. Introduction

Federated learning (FL) has shown potential as a distributed framework that trains a shared global model across multiple clients without sharing their private data. One major challenge in FL is data heterogeneity, where non-IID distributions across clients lead to biased local updates. To address this, researchers propose personalized federated learning (pFL) methods, which allow each client to train a personalized model that adapts to its local data distribution. While pFL can mitigate distributional differences across clients, it generally assumes the availability of sufficient local data [1]. However, in practice, clients typically possess limited local samples due to high data collection costs or naturally scarce data. This raises the following question: How can effective pFL be realized under few-shot conditions?

One promising direction lies in leveraging the hierarchical nature of neural representation, which is inspired by the hierarchical processing mechanism of the human brain. In the human brain, lower-level regions, such as the primary visual cortex, are responsible for extracting general perceptual features like edges and textures, whereas higher-level regions, such as the inferotemporal cortex, handle abstract representations and object identities [2]. Drawing a functional analogy to Convolutional Neural Networks (CNNs), we explicitly map the perceptual processing of lower-level brain regions to the shallow layers of the network, and the representational processing of higher-level brain regions to the deep layers. This structural separation allows humans to rapidly generalize from limited experience, a key advantage in few-shot scenarios [3]. However, many existing pFL approaches focus on model-level personalization [4,5], where aggregation weights are calculated based on the parameter or loss distances of the entire model. Although Ma et al. [6] proposed a layer-wise aggregation framework, they treat all layers equally without distinguishing between generalization in shallow layers and personalization in deep ones [7]. More critically, such methods typically rely on parameter distance or local loss to determine aggregation weights. Under few-shot heterogeneity, local models are prone to overfitting, rendering parameter distance an unreliable proxy for semantic similarity. Consequently, these distance-based layer-wise aggregators fail to capture the true correlation between clients, leading to suboptimal personalization. These limitations highlight the need for a brain-inspired hierarchical pFL framework that can explicitly model and utilize the hierarchical structure, enabling more fine-grained and effective personalization in few-shot settings.

In parallel, another strategy to alleviate this problem is introducing representation-level inductive bias. In few-shot scenarios, these biases guide models to learn more effective data representations with limited samples. Recently, several methods have incorporated different forms of representation-level inductive bias to address the aforementioned challenges. FedFSL-Adv [8] introduces adversarial invariance bias to align data distributions across clients to construct a shared discriminative feature space, where a global classifier is used to perform few-shot classification. In contrast, pFedFSL [9] adopts prototypical networks with inductive bias to construct a discriminative feature space on each client and generates class-wise prototypes, using the distance between query samples and prototypes for classification. However, their inductive bias approaches rely on rigid assumptions—adversarial learning assumes feature robustness through fixed perturbation patterns, while prototypical networks assume classes form compact clusters around fixed prototypes. In federated learning scenarios with high data heterogeneity, distribution shifts across clients violate these assumptions, hindering the alignment and stability of learned representations [10], thereby limiting the performance of such methods [11]. Therefore, there is a need for more adaptive inductive bias approaches that can better handle the complexities of federated few-shot learning scenarios.

The above analysis reveals two basic limitations in the current pFL research under few-shot conditions. Firstly, while the hierarchical nature of neural representation offers a promising path for personalization in few-shot scenarios, most existing pFL methods still rely on model-level aggregation and fail to distinguish the different roles of shallow and deep layers. This limits their ability to balance generalization and personalization in a fine-grained manner. Secondly, recent works attempt to enhance few-shot performance through inductive biases, such as adversarial invariance and prototypical assumptions. However, these methods rely on rigid inductive assumptions that often break down under the distributional shifts typical in federated settings. These limitations call for a hierarchical pFL framework that both explicitly models the layered structure of neural networks and introduces adaptive inductive biases to better address the challenges of federated few-shot learning.

To address these challenges, we propose a novel pFL framework called pFedH2A. Inspired by the hierarchical processing mechanism of the human brain [2], we propose a dual-branch hypernetwork (DHN) designed to facilitate hierarchical personalized aggregation. By heterogeneously generating weights for shallow and deep layers, the DHN enables the model to explicitly decouple and capture the perceptual and representational information embedded in the hierarchical structure of neural networks. In addition, inspired by relation networks [12], we construct an adaptive similarity function for each client in our framework. This function enables structure-independent few-shot class discrimination by learning to measure whether a given pair of samples belongs to the same class, making it adaptable to heterogeneous federal environments. While validated on standard benchmarks, the proposed framework holds significant potential for real-world applications requiring strict privacy, such as medical imaging diagnosis and industrial defect detection. In these trust-critical scenarios, the protection of client identity is a primary concern, as it is encoded to guide personalization. To address this risk, pFedH2A is designed to be compatible with standard privacy-preserving measures. Specifically, protocols such as Secure Aggregation or Local Differential Privacy can be integrated to prevent identity leakage without compromising model utility. The contributions of the paper are summarized as follows:

1. We propose a novel pFL framework pFedH2A. It simulates the division of labor mechanism in different regions of the human brain when processing perception and representation information, and performs personalized aggregation of hierarchical modules in neural networks.
2. We design a dual-branch hypernetwork (DHN) that moves beyond the monolithic parameter generation of conventional hypernetworks. By explicitly decoupling the generation process into perceptual and representational streams, DHN enables fine-grained, layer-adaptive aggregation that balances generalization and personalization, a capability lacking in single-stream approaches.
3. We design a relation-aware module that learns an adaptive similarity function for each client. Unlike standard methods that rely on fixed distance metrics, this module constructs a learnable metric to determine class membership, enabling effective discrimination in heterogeneous few-shot scenarios.
4. We conduct extensive experiments on three public image classification datasets and demonstrate that pFedH2A outperforms other baseline pFL methods in accuracy under few-shot scenarios.

## 2. Related Work

In this section, we introduce previous research on few-shot learning, personalized federated learning, and hypernetwork in federated learning.

### 2.1. Few-Shot Learning

The landscape of few-shot learning has been extensively explored in various approaches and paradigms, highlighting both the simplicity and complexity inherent in effectively learning from limited data. Wang et al. [13] introduce a transformer-based framework tailored for intelligent fault diagnosis, emphasizing robustness under noisy labels and varying operational conditions, which highlights the importance of model resilience in real-world scenarios. Similarly, Yuan et al. [14] develop a complex-valued graph classification framework utilizing a graph transformer to classify space targets in ISAR images, effectively preserving phase information crucial for accurate classification in few-shot settings.

Meta-learning, as an efficient learning strategy, can effectively address scenarios with sparse data. Chen et al. [15] investigate the effectiveness of simple meta-learning over pre-trained classification models. Their analysis reveals nuanced trade-offs between meta-learning objectives and traditional classification objectives, shedding light on the conditions under which meta-learning can be beneficial in few-shot scenarios.

However, meta-learning is not the only approach to addressing the issue of data sparsity. Tian et al. [16] challenge the conventional emphasis on meta-learning by demonstrating that a straightforward approach learning a robust embedding via supervised or self-supervised methods followed by training a linear classifier can outperform more complex meta-learning algorithms. This finding suggests that the quality of the embedding space plays a crucial role in few-shot classification, prompting a reconsideration of the benchmarks and the role of meta-learning in few-shot learning.

In addition, Zhang et al. [17] propose SONO, a method utilizing Second-Order Neural Ordinary Differential Equations to improve cross-modal few-shot learning, demonstrating superior performance across multiple datasets. Furthermore, the scope of representation learning is expanding towards adaptive pattern mining within distributed frameworks. Recent research demonstrates that integrating AI-driven pattern recognition with cloud infrastructure enables robust intelligence in dynamic environments [18]. This perspective supports the shift towards relation-aware methodologies that can adaptively discern patterns from limited data, aligning with the core motivation of our approach. Moreover, comprehensive surveys on few-shot class-incremental learning provide critical insights into adapting to new classes with limited data [19]. These works align closely with our focus on dynamic scenarios, bridging the gap between static few-shot benchmarks and continuous learning environments. Collectively, these studies illustrate the breadth of few-shot learning methodologies, emphasizing their critical role in enabling effective learning with scarce data in various fields.

### 2.2. Personalized Federated Learning

Federated learning has emerged as a promising approach for training machine learning models across multiple clients without the need to share raw data. One key aspect explored in the recent literature is personalized federated learning (pFL), where the goal is to tailor the global model to individual clients’ needs while maintaining privacy and efficiency [20].

To address the challenge of statistical diversity among clients, diverse algorithmic strategies have been proposed. One prominent direction involves decoupling personalized model optimization from global model learning, such as pFedMe, which utilizes Moreau envelopes to regularize local updates [21]. Addressing heterogeneity from a representation perspective, Collins et al. exploited shared global feature representations coupled with unique local heads for each client [22]. Similarly, adaptive local aggregation methods like FedALA have been developed to capture client-specific information by dynamically adjusting the aggregation of the global model [23]. Another influential approach involves model-agnostic meta-learning, which seeks an initial shared model that can be rapidly adapted to the local datasets of individual clients [24].

Beyond algorithmic design, theoretical foundations and system properties have been extensively studied. Lower bounds and optimal algorithms have been established, shedding light on the communication and local oracle complexity of pFL [25]. Furthermore, fairness and robustness are critical considerations; frameworks like Ditto have been integrated to address poisoning attacks while ensuring uniform performance [26]. Complementary to these robustness efforts, establishing trustworthy IoT infrastructures further requires efficient secure aggregation protocols that minimize computational overhead while defending against privacy breaches and evolving cyber threats [27].

In terms of practical deployment, pFL has been applied in various domains, such as network traffic anomaly detection [28] and intelligent IoT applications [20]. However, real-world implementation faces distinct challenges. Recent studies on Raspberry Pi platforms for IoT 6G applications have highlighted the deployment constraints and resource limitations inherent in these environments [29], emphasizing the need for efficient personalization strategies that can operate under such benchmarks.

Overall, while pFL remains an active research area, significant challenges persist. The intersection of federated learning and AI-generated content (AIGC) represents a rapidly evolving frontier, with recent surveys offering valuable perspectives on future extensions towards generative capabilities [30]. Nevertheless, regarding the issue of data scarcity, how to maintain efficient learning capabilities with limited data on the client side remains an open problem that needs to be addressed.

### 2.3. Hypernetwork in Federated Learning

The application of hypernetworks within federated learning (FL) has garnered significant attention in recent research, primarily due to their capacity to enhance personalization, address heterogeneity, and improve model stability across distributed clients. Several studies have explored hypernetwork-based approaches to tackle the inherent challenges of FL, such as data disparities, resource constraints, and privacy concerns.

Personalized federated learning leveraging hypernetworks has been prominently investigated. Shamsian et al. [28] introduced pFedHN, a hypernetwork-based method designed to generate personalized models for individual clients, effectively accounting for data heterogeneity while reducing communication costs. Building upon this, Yang et al. [31] proposed HyperFed, a hypernetwork-driven federated learning framework tailored for multi-institutional CT imaging. This approach aims to mitigate domain shift issues and privacy concerns by enabling personalized model training without centralized data collection. Similarly, Guo et al. [32] developed HyperFL, which utilizes hypernetworks to generate local model parameters, with only hypernetwork parameters being uploaded to the server, thereby enhancing privacy protection.

The versatility of hypernetworks extends to addressing client heterogeneity and resource constraints. Shin et al. [33] introduced HypeMeFed, combining multi-exit network architectures with hypernetwork-based weight generation to support clients with varying capabilities. In resource-scarce environments, Zhang et al. [34] proposed RecipFL, employing a server-side graph hypernetwork to incentivize device participation and improve accuracy across devices with different resource levels.

Finally, the application of hypernetworks in physics-driven and industrial contexts also demonstrates their broad utility. Yang et al. [35] employed hypernetwork-based, physics-driven personalized FL for CT imaging, aiming to learn invariant features across diverse data distributions. Zhang et al. [36] applied hypernetwork models for industrial anomaly detection, emphasizing model stability and rapid knowledge transfer in dynamic environments.

In summary, the integration of hypernetworks into federated learning frameworks has shown promising results in personalization, resource efficiency, and heterogeneity support. These studies collectively highlight hypernetworks as a versatile and powerful tool to address the multifaceted challenges of federated learning across various domains.

## 3. Proposed Method

**Figure 1:**
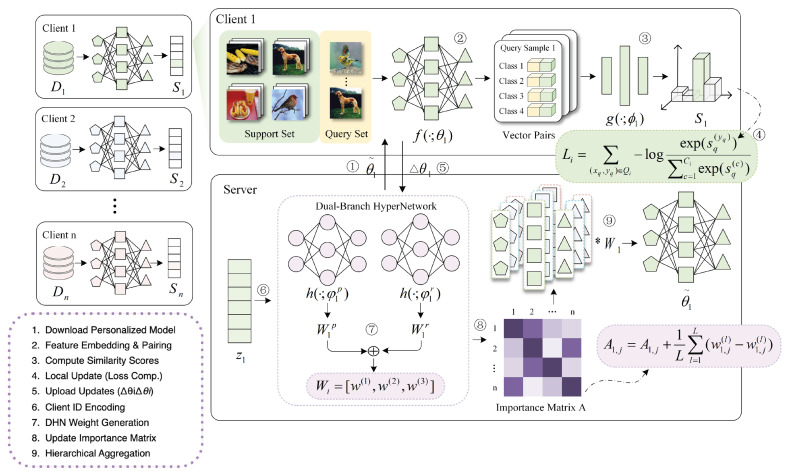
Architecture of pFedH2A. The workflow contains the following steps: (1) The client obtains the personalized model from the server; (2) maps the support and query sets into embedding vectors and constructs sample pairs; (3) computes similarity scores using a similarity function; (4) calculates the local loss based on the scores and updates the model; and (5) uploads the parameter updates to the server. (6) The server encodes the client identity into an embedding vector; (7) feeds it into the dual-branch hypernetwork to generate perception and representation weights, which are fused into hierarchical aggregation weights; (8) updates the importance matrix based on the aggregation weights and selects reference clients; and (9) performs hierarchical aggregation over reference models using the weights to generate the updated personalized model.

In this section, we present the design of pFedH2A, which aims to achieve effective personalization in FL under few-shot settings. The overall architecture of pFedH2A is illustrated in Figure 1. The description is organized into three parts: problem formulation, hierarchical personalized aggregation via the dual-branch hypernetwork, and relation-aware personalized federated learning for few-shot scenarios.

### 3.1. Problem Formulation

In this section, we define the problem of personalized model training, including the local dataset structure and the optimization objective.

Assume there are *U* clients, where each client i∈[1,U] holds a local dataset $D_{i}$ of size $m_{i}$. The local dataset is defined as

(1) $$D_{i}=\left\{\left(x_{j}^{i},y_{j}^{i}\right)|j=1,\ldots ,m_{i}\right\},$$

where $x_{j}^{i}$ denotes the input sample and $y_{j}^{i}$ is the corresponding class label. The total number of data samples across all clients satisfies $\sum_{i=1}^{U}m_{i}=M$, where *M* represents the overall dataset size.

In few-shot scenarios, each client *i* participates in a $C_{i}$-way $K_{i}$-shot task, where $C_{i}$ is the number of sampled classes and $K_{i}$ is the number of labeled samples per class. During each training iteration, client *i* randomly selects $C_{i}$ classes from $D_{i}$, sampling $K_{i}$ labeled samples per class to construct the support set $S_{i}$:

(2) $$S_{i}=\left\{\left(x_{j}^{i},y_{j}^{i}\right)|j=1,\ldots ,C_{i}\times K_{i}\right\}.$$

The remaining labeled samples from the selected $C_{i}$ classes are used to form the query set $Q_{i}$, which shares the same structure as $S_{i}$.

Let $\theta_{i}$ denote the local model parameters of client *i*. The objective is to learn a set of personalized models $\left\{\theta_{1},\ldots ,\theta_{U}\right\}$ by minimizing the sum of local empirical losses across clients. In few-shot settings, the loss is typically computed on the query set after model adaptation on the support set:

(3) $$\underset{\left\{\theta_{1},...,\theta_{U}\right\}}{\mathrm{argmin}}\sum_{i=1}^{U}\frac{m_{i}}{M}L\left(\theta_{i},Q_{i}\right).$$

### 3.2. Hierarchical Personalized Aggregation via the Dual-Branch Hypernetwork

**Figure 2:**
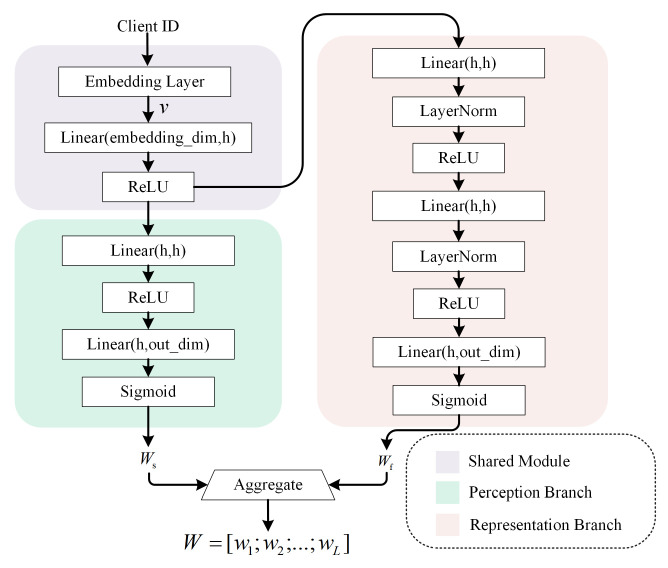
Architecture of DHN.

Inspired by the functional similarity between the human brain and artificial neural networks in hierarchical feature processing [37], we propose a Dual-branch HyperNetwork (DHN). The DHN distinguishes the roles of shallow and deep neural layers in handling perceptual and abstract representational information, respectively. By decoupling the modeling of local personalization and global generalization, DHN facilitates the generation of hierarchical aggregation weights. As illustrated in Figure 2, the DHN comprises three modules: a shared module, a perception branch, and a representation branch.

In order to support two branches to generate more accurate and adapted aggregation weights based on client differences, we set up a pre-sharing module to extract a client-specific representation, rather than directly feeding the client identifier into the two hypernetwork branches. Specifically, the module first maps the discrete client identifier to a dense embedding vector $v_{i}$ through an embedding layer. Then, this embedding is transformed by a fully connected layer followed by a ReLU activation to obtain the representation vector $z_{i}$, as shown in Equation (4).

(4) $$z_{i}=ReLU\left(FC\left(v_{i}\right)\right)$$

Each branch is implemented as a hypernetwork that takes the client-specific vector $z_{i}$ as input. Specifically, the perception branch $h^{p}\left(\cdot \right)$ and the representation branch $h^{r}\left(\cdot \right)$ generate hierarchical aggregation weights as follows:

(5) $$W_{i}^{p}=h^{p}\left(z_{i};\varphi_{i}^{p}\right),\;W_{i}^{r}=h^{r}\left(z_{i};\varphi_{i}^{r}\right)$$

where $\varphi_{i}^{p}$ and $\varphi_{i}^{r}$ denote the parameters of the perception and representation branches, respectively, and $W_{i}^{p}$ and $W_{i}^{r}$ are the corresponding aggregation weights.

Mechanistically, the functional distinction between the two branches is structurally enforced by their architectural designs. As shown in Figure 2, the perception branch $h^{p}$ employs a shallow architecture consisting of only two linear layers without normalization. This limited complexity imposes a structural regularization, compelling the branch to function as a stabilizer that generates smooth and stable weight distributions capturing shared general knowledge. In contrast, the representation branch $h^{r}$ features a deeper architecture enhanced with Layer Normalization (LayerNorm). The increased depth provides the necessary capacity to model complex non-linear relationships, while LayerNorm facilitates fine-grained feature calibration. This design empowers the branch to act as a selector, generating highly discriminative weights that selectively activate layers critical for semantic interpretation. Consequently, the final fused weight $W_{i}$ dynamically combines this stable foundation with selective personalization.

To adaptively fuse the contributions of these two branches, thereby balancing the modeling of global shared and client-specific information, we introduce a dynamic fusion coefficient $\alpha_{i}$ to obtain the final aggregation weights $W_{i}$:

(6) $$W_{i}=\left(1-\alpha_{i}\right)\cdot W_{i}^{p}+\alpha_{i}\cdot W_{i}^{r}$$

where $\alpha_{i}$ is designed to adaptively control the fusion ratio between the perception and representation branches, as shown in Equation (7).

(7) $$\alpha_{i}=\sigma \left(\gamma \cdot \left(\frac{1}{\left|\Theta_{i}\right|}\sum_{\theta_{j}\in \Theta_{i}}{∥\theta_{i}-\theta_{j}∥}_{2}^{2}\right)\right)$$

where σ(·) denotes the sigmoid function to normalize the output to the range (0,1), γ is a scaling factor that controls the sensitivity to distributional deviation, $\theta_{i}$ denotes the model parameters of the target client *i*, $\Theta_{i}$ represents the set of model parameters corresponding to the selected reference clients (i.e., $\Theta_{i}={\left\{\theta_{n}\right\}}_{n=1}^{N}$, including client *i* itself), and $\theta_{j}\in \Theta_{i}$ denotes the parameters of a specific reference client.

Since $W_{i}$ is a hierarchical aggregation weight matrix, it is represented in Equation (8) as an explicit matrix of shape $R^{N\times L}$, where *N* is the number of selected reference clients (including client *i*) and *L* is the number of model layers. In this matrix, $w_{n}^{\left(l\right)}$ denotes the aggregation weight assigned to the *l*-th layer of the model from the *n*-th client. Each column corresponds to a specific model layer, and each row corresponds to a specific client.

(8) $$\begin{array}{c} W_{i}=\left[w^{\left(1\right)},w^{\left(2\right)},\ldots ,w^{\left(L\right)}\right]=\left[\begin{array}{cccc} w_{1}^{\left(1\right)} & w_{1}^{\left(2\right)} & \ldots & w_{1}^{\left(L\right)}\\ w_{2}^{\left(1\right)} & w_{2}^{\left(2\right)} & \ldots & w_{2}^{\left(L\right)}\\ \vdots & \vdots & \ddots & \vdots \\ w_{N}^{\left(1\right)} & w_{N}^{\left(2\right)} & \ldots & w_{N}^{\left(L\right)} \end{array}\right] \end{array}$$

To strictly enforce the normalization constraint, we apply a Softmax projection to the weights along the client dimension. Specifically, the normalized weight $w_{n}^{\left(l\right)}$ for the *l*-th layer of the *n*-th reference client is computed from the raw logits ${\tilde{w}}_{n}^{\left(l\right)}$ as

(9) $$w_{n}^{\left(l\right)}=\frac{\exp\left({\tilde{w}}_{n}^{\left(l\right)}\right)}{\sum_{k=1}^{N}\exp\left({\tilde{w}}_{k}^{\left(l\right)}\right)}$$

This projection ensures that the generated weights form a valid probability distribution satisfying the following constraint:

(10) $$\sum_{n=1}^{N}w_{n}^{\left(l\right)}=1,\forall l=1,2,\ldots ,L$$

To enhance aggregation efficiency and reduce communication overhead, we introduce an importance matrix A to record the aggregation performance between clients. Specifically, for a target client *i*, the server selects the top-*N* most relevant clients (including *i* itself) to form a reference client set based on the matrix. This client selection strategy reduces the number of uploaded models per round, thereby alleviating communication costs. The importance score $A_{i,j}$ is dynamically updated using the hierarchical aggregation weights $W_{i}$, which encourages collaboration among clients with similar data distributions. Specifically, we quantify the value of a peer client *j* by computing the average difference between its assigned weight $w_{i,j}^{\left(l\right)}$ and the client’s self-weight $w_{i,i}^{\left(l\right)}$ across all layers, as shown in Equation (11):

(11) $$A_{i,j}=A_{i,j}+\frac{1}{L}\sum_{l=1}^{L}\left(w_{i,j}^{\left(l\right)}-w_{i,i}^{\left(l\right)}\right)$$

The rationale behind this update rule is grounded in the concept of relative information gain. This metric captures the net contribution of a reference client relative to the local model’s self-confidence. A positive value implies that client *j* provides valuable features that the local model *i* currently lacks or values more than its own parameters, thereby increasing the priority of client *j* in future selections. Furthermore, the number of selected reference clients, *N*, serves as a critical hyperparameter balancing knowledge diversity and noise. A small *N* ensures that only the most relevant models are aggregated, minimizing negative transfer, while a larger *N* increases the diversity of the feature space but risks introducing noise. Therefore, *N* acts as a selective filter to ensure high-quality collaboration.

Finally, utilizing the identified reference client set $\Theta_{i}$ and the generated hierarchical weight matrix $W_{i}$, the hierarchical aggregation function A computes the updated personalized model ${\tilde{\theta }}_{i}$ as follows:

(12) $${\tilde{\theta }}_{i}=A\left(W_{i};\Theta_{i}\right)=\left\{{\tilde{\theta }}_{i}^{\left(1\right)},{\tilde{\theta }}_{i}^{\left(2\right)},\ldots ,{\tilde{\theta }}_{i}^{\left(L\right)}\right\}$$

where each layer is aggregated as

(13) $${\tilde{\theta }}_{i}^{\left(l\right)}=\sum_{n=1}^{N}w_{n}^{\left(l\right)}\theta_{n}^{\left(l\right)},\forall l=1,2,\ldots ,L$$

Through this layer-wise aggregation mechanism, pFedH2A constructs a personalized model ${\tilde{\theta }}_{i}$ that effectively balances the global knowledge and client-specific patterns. The detailed training and update procedures for these components are elaborated in the following section.

### 3.3. Relation-Aware Personalized Federated Learning for Few-Shot Scenarios

To enable effective federated personalization in few-shot environments, we propose a relation-based dual-module architecture for each client. This design comprises a feature encoder module $f(\cdot ;\theta_{i})$ with parameters $\theta_{i}$ and a similarity metric module $g(\cdot ;\phi_{i})$ with parameters $\phi_{i}$. The encoder extracts latent representations from input samples, while the similarity module measures the relation between query and support representations to guide class predictions.

To obtain the embedding representations needed for similarity comparison, the feature encoder maps each support and query sample into a shared latent space as follows:

(14) $$v_{s}=f\left(x_{s};\theta_{i}\right),\;v_{q}=f\left(x_{q};\theta_{i}\right)$$

where $x_{s}\in S_{i}$ and $x_{q}\in Q_{i}$ represent individual samples. For each class $c\in \left\{1,\ldots ,C_{i}\right\}$, we compute a fused support vector $v^{\left(c\right)}$ by aggregating the feature vectors of all support samples labeled as class *c*. Specifically,

(15) $$v^{\left(c\right)}=\frac{1}{K_{i}}\sum_{\left(x_{s},y_{s}\right)\in S_{i}}I\left(y_{s}=c\right)\cdot v_{s}$$

where $y_{s}$ is the ground-truth label of support sample $x_{s}$, I(·) is the indicator function.

Then, for each query feature vector $v_{q}$, we compute its similarity with each class prototype $v^{\left(c\right)}$ by concatenating the two vectors and feeding the result into a similarity metric module $g(\cdot ;\phi_{i})$.

(16) $$s_{q}^{\left(c\right)}=g\left(\left[v_{q},v^{\left(c\right)}\right];\phi_{i}\right),\;c\in 1,\ldots ,C_{i}$$

where $s_{q}^{\left(c\right)}$ denotes the similarity between the query sample $x_{q}$ and the support set of class *c*. These class-wise similarity scores are subsequently used to determine the predicted class label of the query.

To train the model to produce discriminative similarity scores, we minimize the cross-entropy loss over the predicted scores and the ground-truth labels of query samples within each training iteration:

(17) $$L_{i}=\sum_{\left(x_{q},y_{q}\right)\in Q_{i}}-\log\frac{\exp(g_{\phi_{i}}\left(\left[f_{\theta_{i}}\left(x_{q}\right),v^{\left(y_{q}\right)}\right]\right))}{\sum_{c=1}^{C_{i}}\exp\left(g_{\phi_{i}}\left(\left[f_{\theta_{i}}\left(x_{q}\right),v^{\left(c\right)}\right]\right)\right)}$$

where $E_{T_{i}∼D_{i}}$ denotes the expectation over few-shot episodes $T_{i}$ sampled from client i’s local dataset $D_{i}$, where each episode includes a support set $S_{i}$ and a query set $Q_{i}$. The fused support feature $v^{\left(c\right)}$ is obtained by averaging the embeddings of all support samples belonging to class *c*. In particular, $v^{\left(y_{q}\right)}$ refers to the fused feature vector of the ground-truth class. This softmax-based cross-entropy formulation encourages the model to assign a higher similarity score to the true class than to the others, facilitating accurate classification in the few-shot setting.

Consequently, the client-side loss $L_{i}$ defined in Equation (17) can be used to optimize $\theta_{i}$ and $\phi_{i}$. Specifically, the gradients of $L_{i}$ with respect to these two components can be computed as

(18) $$\nabla_{\theta_{i}}L_{i}=\frac{\partial L_{i}}{\partial \theta_{i}},\;\nabla_{\phi_{i}}L_{i}=\frac{\partial L_{i}}{\partial \phi_{i}}$$

Accordingly, standard gradient descent can be applied as Equation (19), where η represents the local learning rate.

(19) $$\theta_{i}\leftarrow \theta_{i}-\eta \cdot \nabla_{\theta_{i}}L_{i},\;\phi_{i}\leftarrow \phi_{i}-\eta \cdot \nabla_{\phi_{i}}L_{i}$$

To address the challenges posed by data heterogeneity, we adopt distinct optimization strategies for the client models. Since the structure and distribution of local classes vary across clients, enforcing global aggregation on $\phi_{i}$ could impair the ability to learn accurate similarity representations tailored to the local task. In contrast, the feature encoder module parameter $\theta_{i}$ is jointly optimized through local training and global aggregation. This is because $\theta_{i}$ is responsible for mapping the original input into the embedding space, and relying solely on local data for training can lead to overfitting issues.

As described in Section C, the personalized aggregation process is guided by the DHN. Based on Equations (5) and (6), we treat ${\tilde{\theta }}_{i}=A\left(W_{i};\Theta_{i}\right)$ as

(20) $${\tilde{\theta }}_{i}=A\left(\alpha_{i}h^{p}\left(z_{i};\varphi_{i}^{p}\right)+\left(1-\alpha_{i}\right)h^{r}\left(z_{i};\varphi_{i}^{r}\right);\Theta_{i}\right)$$

which indicates that the generation of ${\tilde{\theta }}_{i}$ directly influenced by the $\varphi_{i}^{p}$, $\varphi_{i}^{r}$ and $z_{i}$. Consequently, we realize that the optimization of the client model is transformed into a joint optimization over the DHN parameters and input.

Since $z_{i}$, $\varphi_{i}^{p}$, and $\varphi_{i}^{r}$ serve as the input and parameters of the DHN deployed on the server, federated communication is necessary to support their optimization. To achieve this without directly transmitting local models, the client uploads the parameter increment $△\theta_{i}=\theta_{i}-{\tilde{\theta }}_{i}$ after local training. This approach not only enhances communication efficiency but also helps preserve data privacy. Based on the received parameter increment, the server can then compute the gradients for updating the DHN input and parameters using the chain rule as follows:

(21) $$\begin{array}{cc} \nabla_{z_{i}}L_{i} & ={\left(\nabla_{z_{i}}{\tilde{\theta }}_{i}\right)}^{T}\nabla_{{\tilde{\theta }}_{i}}L_{i}\\ \nabla_{\varphi_{i}^{p}}L_{i} & ={\left(\nabla_{\varphi_{i}^{p}}{\tilde{\theta }}_{i}\right)}^{T}\nabla_{{\tilde{\theta }}_{i}}L_{i}\\ \nabla_{\varphi_{i}^{r}}L_{i} & ={\left(\nabla_{\varphi_{i}^{r}}{\tilde{\theta }}_{i}\right)}^{T}\nabla_{{\tilde{\theta }}_{i}}L_{i} \end{array}$$

Subsequently, these hyperparameters are updated using the gradient information and the received model difference $\Delta \theta_{i}$, where α is the meta-learning rate:

(22) $$\begin{array}{cc} z_{i} & \leftarrow z_{i}-\beta {\left(\nabla_{z_{i}}{\tilde{\theta }}_{i}\right)}^{T}\Delta \theta_{i}\\ \varphi_{i}^{p} & \leftarrow \varphi_{i}^{p}-\beta {\left(\nabla_{\varphi_{i}^{p}}{\tilde{\theta }}_{i}\right)}^{T}\Delta \theta_{i}\\ \varphi_{i}^{r} & \leftarrow \varphi_{i}^{r}-\beta {\left(\nabla_{\varphi_{i}^{r}}{\tilde{\theta }}_{i}\right)}^{T}\Delta \theta_{i} \end{array}$$

### 3.4. Overall Workflow

The collaborative procedures of pFedH2A involve coordination between client-side local training and server-side hierarchical aggregation, which is outlined in Algorithm 1. In each round of communication, the client first downloads the updated personalized model from the server and performs local training to obtain the parameter increments. These increments are uploaded to the server to drive the personalization process. Subsequently, the server executes the hierarchical aggregation, which proceeds in three distinct steps:
**Algorithm 1** pFedH2A Algorithm**Input:** 
Number of selected clients *N*, model layers *L*, local parameters θ and ϕ, DHN parameters $\varphi^{p}$ and $\varphi^{r}$, meta-learning rate α, local learning rate η, dataset $\left\{D_{1},D_{2},\ldots ,D_{U}\right\}$, importance matrix A=diag(1,…,1);**Output:** 
Updated local personalized model parameters $\left\{{\tilde{\theta }}_{1},{\tilde{\theta }}_{2},\ldots ,{\tilde{\theta }}_{U}\right\}$;1:  **for** each communication round *t* **do**2:      **for** each client *i* **do**3:          $W_{i}^{p}=h^{p}\left(z_{i};\varphi_{i}^{p}\right),\;W_{i}^{r}=h^{r}\left(z_{i};\varphi_{i}^{r}\right)$4:          $W_{i}=\left(1-\alpha_{i}\right)\cdot W_{i}^{p}+\alpha_{i}\cdot W_{i}^{r}$5:          $A_{i,j}=A_{i,j}+\frac{1}{L}\sum_{l=1}^{L}\left(w_{i,j}^{\left(l\right)}-w_{i,i}^{\left(l\right)}\right)$6:          Select *N* clients based on *A*7:          Receive $\Theta_{i}=\left\{\theta_{1},\theta_{2},...,\theta_{N}\right\}$8:          ${\tilde{\theta }}_{i}=A\left(W_{i};\Theta_{i}\right)=\left\{{\tilde{\theta }}_{i}^{\left(1\right)},{\tilde{\theta }}_{i}^{\left(2\right)},\ldots ,{\tilde{\theta }}_{i}^{\left(L\right)}\right\}$9:          $\Delta \theta_{i}=\mathrm{ClientTrain}\left({\tilde{\theta }}_{i}\right)$10:        Update $z_{i},\varphi_{i}^{p}$, $\varphi_{i}^{r}$ via Equation (22)11:    **end for**12:**end for**13:**function** ClientTrain(${\tilde{\theta }}_{i}$)14:    Set $\theta_{i}={\tilde{\theta }}_{i}$15:    **for** each epoch *e* **do**16:        **for** mini-batch $T_{i}\subset D_{i}$ **do**17:           Split $T_{i}$ into support set $S_{i}$ and query set $Q_{i}$18:           $v_{s}=f\left(x_{s};\theta_{i}\right),\;v_{q}=f\left(x_{q};\theta_{i}\right)$19:           $s_{q}^{\left(c\right)}=g\left(\left[v_{q},v^{\left(c\right)}\right];\phi_{i}\right),\;c\in 1,\ldots ,C_{i}$20:           $\theta_{i}\leftarrow \theta_{i}-\eta \cdot \nabla_{\theta_{i}}L_{i},\;\phi_{i}\leftarrow \phi_{i}-\eta \cdot \nabla_{\phi_{i}}L_{i}$21:        **end for**22:    **end for**23:    **return** $\Delta \theta_{i}=\theta_{i}-{\tilde{\theta }}_{i}$24:**end function**

1. Weight Generation: The server encodes the target client identity into $z_{i}$ and feeds it into the DHN to generate perception and representation weights. These are fused via $\alpha_{i}$ to produce the final hierarchical weight matrix $W_{i}$.
2. Reference Selection: The importance matrix *A* is dynamically updated based on the generated weights to reflect the current correlation between clients. Using this updated matrix, the server identifies a set of reference clients $\Theta_{i}$ to participate in the aggregation.
3. Hierarchical Aggregation: The personalized model ${\tilde{\theta }}_{i}$ is constructed by aggregating the parameters of the selected reference clients layer-by-layer, using the specific weights assigned in $W_{i}$ to distinctively combine shallow and deep features.

Beyond the algorithmic workflow, we further characterize the proposed framework from three critical dimensions: optimization stability, system scalability, and privacy compatibility.

First, regarding optimization stability, the training process is structured as a robust bilevel optimization problem. The inner loop optimizes the client-specific parameters $\theta_{i}$ and $\phi_{i}$ with the local learning rate η to minimize the loss $L_{i}$. Conversely, the outer loop updates the hypernetwork parameters $\varphi^{p},\varphi^{r}$ and client embeddings $z_{i}$ using the parameter increments $\Delta \theta_{i}$ and a meta-learning rate β. This two-timescale strategy prevents overreaction to noisy gradients, facilitating rapid local adaptation while ensuring robust aggregation over a longer horizon.

Second, the framework ensures system scalability by minimizing operational overhead. Regarding communication, the generation of hierarchical weights by the DHN is executed entirely on the server, meaning clients upload only the parameter increments $\Delta \theta_{i}$ of the backbone model. Regarding computation, the DHN employs a lightweight MLP-based architecture, rendering its forward pass negligible compared to the backward propagation of the CNN backbone. Consequently, pFedH2A achieves effective personalization with minimal resource consumption.

Third, the design exhibits inherent privacy compatibility suitable for secure real-world deployments. Specifically, the framework allows for the integration of Local Differential Privacy by injecting noise into the embedding vector $z_{i}$ before transmission to prevent identity leakage. As a continuous function approximator, the HyperNetwork remains robust to such perturbations. Additionally, standard Secure Aggregation protocols can be employed to mask the transmitted parameter increments $\Delta \theta_{i}$, ensuring that the server aggregates updates without inspecting individual client models.

## 4. Experiment

In this section, we describe the experimental setup and evaluate the performance of pFedH2A through various methods, such as comparative experiments and ablation experiments. In Section 4.1, we introduce the experimental dataset, data allocation methods, baseline methods for comparison, and some implementation details. In Section 4.2, we presents a thorough evaluation of pFedH2A from multiple perspectives. First, we compare its performance with baseline methods under various settings. Then, we conduct ablation studies to examine the contribution of key components. To further understand the behavior of the proposed method, we perform cluster sensitivity analysis, analyze the evolution of client importance, and visualize the hierarchical weight fusion process.

### 4.1. Experimental Setup

**Dataset.** We evaluate the performance of the proposed method on three public datasets: MNIST, CIFAR-10, and CIFAR-100. MNIST, a classic baseline for handwritten digit recognition, consists of 70,000 grayscale images of 28 × 28 pixels, evenly distributed across 10 digit classes (0–9). CIFAR-10 targets general object classification tasks, comprising 60,000 RGB images of 32 × 32 pixels across 10 basic categories, including airplanes, automobiles, and birds. As an extended version, CIFAR-100 maintains the same image resolution but increases the number of categories to 100, thus raising the difficulty of the classification task.

To meet the requirements of few-shot scenarios and simulate the differences in data heterogeneity in practice, we expand the few-shot setting of the general C-way K-shot approach and propose two allocation methods to process the dataset.

- Random heterogeneous allocation: This method constructs heterogeneous client data distributions through a randomization mechanism. Specifically, the number of classes $C_{i}$ contained in each client’s local dataset is randomly selected from [C−1,C+1]. Then, the number of samples $K_{i}$ is randomly chosen from [K−2,K+2] for each selected class. This method creates a unique task space for each client and enhances data heterogeneity.
- Cluster-sharing allocation: This method partitions all clients into groups, with each group assigned *C* target classes to ensure that clients within the same group share a similar task structure. Based on this, training samples are drawn for each client within the cluster using a Dirichlet distribution. This method enables task space sharing within clusters while preserving a certain degree of individual diversity.

Specifically, both methods ensure that for the client *i*, the number of samples in the training and testing sets satisfies $C_{i}\times K_{i}$ and $C_{i}\times 2K_{i}$, respectively.

**Baseline.** We use a systematic comparative experimental framework to compare pFedH2A with five baseline methods, including a classic FL method (FedAvg) and four representative pFL methods (FedFomo, FedBN, pFedHN, pFedFSL).

- FedAvg [38] is a classic FL method that obtains a global model by weighted averaging the local models sent by clients.
- FedFomo [5] learns the optimal weighted combination for each personalized model by calculating the importance between client models.
- FedBN [39] proposed a scheme for cross-client parameter sharing, where each client’s Batch Normalization(BN) layer is updated locally, and other layers are aggregated based on the FedAvg.
- pFedHN [28] generates personalized models directly for each client through a hypernetwork deployed on the server.
- FedFSL [8] is a FedAvg-based method by combines meta-learning techniques such as MAML with adversarial objectives to encourage the construction of a unified and discriminative feature space across clients.
- pFedFSL [9] is a pFL framework in few-shot learning scenarios that enhances the ability to handle sparse data by constructing prototypes for local data.

The experimental setup adheres to the principle of fairness. All baseline methods use the data allocation methods described above and are implemented following the experimental settings specified in their papers.

**Configuration.** For the above methods, we set the number of clients to 30, the communication round to 500, the local training round to 5, and the optimizer to select SGD with a learning rate of 0.01. For methods using hypernetworks (pfedhn and our method), the hypernetwork learning rate is uniformly set at 0.005. For our method, the coefficient γ is set to 0.5.

To ensure a fair evaluation, all methods (including baselines and pFedH2A) utilize the same Convolutional Neural Network (CNN) as the backbone architecture. This ensures that the model capacity and parameter counts are identical across all comparisons. Furthermore, we maintain consistent communication protocols across methods to ensure comparable resource usage.

In addition, we simulate the client and server of the FL framework on devices equipped with Intel Core i7-10700k processors (Intel Corporation, Santa Clara, CA, USA), NVIDIA GeForce RTX 3090 graphics cards (NVIDIA Corporation, Santa Clara, CA, USA), and 16 GB RAM. All methods are implemented in PyTorch (version 1.12.1).

### 4.2. Experimental Results and Analysis

#### 4.2.1. Comparison Experiment

To verify the effectiveness of pFedH2A in few-shot scenarios, we conduct comparative experiments with five baseline methods on three image classification datasets. Furthermore, to evaluate the robustness of various methods under different task complexities and sample scarcity, we selected four few-shot learning settings for experimentation. Table 1 and Table 2 show the comparative experimental results under random heterogeneous allocation and cluster-sharing allocation, respectively. It is noted that the reported values in these tables represent the average accuracy across all participating clients. While individual performance may vary due to the heterogeneity of local data distributions, the average metric serves as a primary indicator of the overall effectiveness of the framework.

The experimental results clearly highlight the superior performance of the proposed method across all datasets and configurations. It consistently outperforms all baseline approaches, achieving the highest accuracy in every scenario. For example, under the most challenging setting (CIFAR-100, 20-way 5-shot), it achieves 27.17% accuracy under random allocation and 38.66% under cluster sharing—both surpassing the second-best method by margins of 3.72% and 4.6%, respectively.

The impact of data allocation strategy is also evident. Table 1 adopts random heterogeneous allocation, where each client has a different number of classes and the number of samples in each class is also uncertain, which enhances the heterogeneity of the data. The cluster-sharing allocation in Table 2 divides clients into several clusters, with each cluster sharing the same target class, ensuring the similarity of task structures for each client. Therefore, the performance of each method in Table 2 is usually better than in Table 1.

Moreover, the inherent difficulty level of each dataset and few-shot configuration further influences model accuracy. Datasets like MNIST, with simple grayscale images and distinct inter-class boundaries, yield consistently higher accuracy than CIFAR-10 or CIFAR-100, which feature complex textures and high inter-class similarity. Additionally, performance improves with increasing shot numbers, as more data supports better generalization. However, increasing the number of classes typically reduces accuracy due to heightened classification difficulty. Notably, our method exhibits stability even in extreme few-shot scenarios, attributable to its effective modeling of inter-sample relationships.

#### 4.2.2. Ablation Experiment

To further validate the effectiveness of each component in pFedH2A, we designed ablation experiments on the MNIST, CIFAR-10, and CIFAR-100 datasets. Specifically, by removing or replacing components from pFedH2A, we constructed the following simplified variants:

- Variant 1: Randomly select reference clients during the aggregation process instead of using the importance matrix for model selection.
- Variant 2: Remove the hierarchical aggregation structure and adopt a overall model aggregation approach.
- Variant 3: Remove the dual-branch hypernetwork design and generate aggregation weights using a single-branch hypernetwork.
- Variant 4: Replace the adaptive relation-aware similarity module with a standard fixed Euclidean distance metric (similar to Prototypical Networks) to evaluate the benefit of the learnable metric.
- Variant 5: Replace the adaptive fusion coefficient $\alpha_{i}$ with a fixed value (set to 0.5) to evaluate the necessity of the dynamic fusion mechanism.

As shown in Table 3, pFedH2A consistently outperforms all variants across different datasets and few-shot settings, with larger performance margins on more complex datasets.

Further analysis reveals the individual impact of each module. Variant 1 replaces importance-based client selection with random sampling, resulting in the most significant accuracy drop, underscoring the necessity of the importance matrix for stable and effective reference aggregation. Variant 2, which removes the hierarchical aggregation structure, performs slightly better than Variant 1 on complex datasets but still trails the full model, indicating that hierarchical design improves personalization granularity and model expressiveness. Variant 3, which eliminates the dual-branch hypernetwork, shows competitive performance on CIFAR-100 but suffers substantial drops on MNIST and CIFAR-10, suggesting its particular importance for handling heterogeneous and simpler data distributions.

Furthermore, the comparison with Variant 4 highlights the contribution of the relation-aware module. The clear performance gap between pFedH2A and Variant 4 demonstrates that a learnable, client-specific similarity function is significantly more effective than rigid distance-based metrics (such as Euclidean distance) in capturing complex class relationships in federated heterogeneous environments. Similarly, the results of Variant 5 validate the necessity of the adaptive fusion mechanism. By replacing the dynamic fusion coefficient with a fixed value, Variant 5 exhibits a notable decline in accuracy, particularly on CIFAR-10 and CIFAR-100. This confirms that a static fusion strategy cannot adequately balance the trade-off between perception and representation across different layers, whereas our adaptive approach dynamically optimizes this balance.

In general, these results confirm that the importance matrix, hierarchical aggregation, dual-branch hypernetwork, relation-aware module, and adaptive fusion mechanism each play a vital role in enhancing the performance of pFedH2A.

#### 4.2.3. Cluster Sensitivity Analysis

To evaluate the impact of different clustering granularities on the performance of pFedH2A, we conduct experiments on three commonly used image classification datasets: MNIST, CIFAR-10, and CIFAR-100. The number of clusters is set to {1,3,5,10,15,30}, where a larger number indicates finer-grained personalization. For each setting, we repeat the experiment five times and report both the maximum (Max) and average (Avg) accuracy to assess the best-case performance and overall stability. All experiments are conducted under a 5-way 5-shot few-shot setting. In addition, we include a baseline method with a randomly generated heterogeneous method.

As shown in Table 4, increasing the number of clusters generally leads to a decline in accuracy across all datasets. This is mainly because finer-grained clustering increases heterogeneity among clients and reduces the effectiveness of knowledge sharing.

Despite this overall trend, the structured cluster-sharing approach consistently outperforms the random heterogeneous baseline, even under extreme settings with 15 or 30 clusters. This indicates that a clustering strategy can alleviate the negative impact of over-fragmentation by enabling more stable aggregation and more meaningful reference selection. These results demonstrate the importance of incorporating structural priors into client grouping, which helps achieve more robust personalized federated learning.

#### 4.2.4. Importance Evolution Analysis

**Figure 3:**
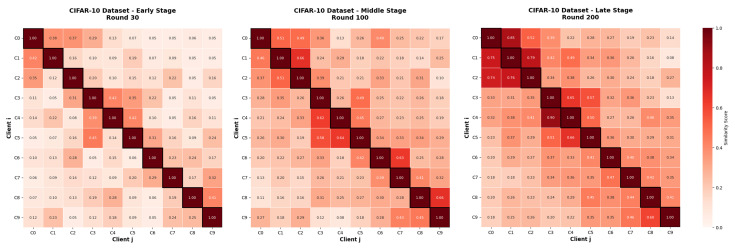
Importance scores between 10 clients at early (round 30), middle (round 100), and late (round 200) training stages on CIFAR-10 with few-shot tasks and random heterogeneous allocation.

To analyze the evolution of client importance during training, we conduct experiments on CIFAR-10 using a 5-way 5-shot few-shot setting, and adopt a random heterogeneous allocation strategy to simulate a heterogeneous data distribution in 10 clients. Figure 3 show the dynamically updated importance scores between clients at different training stages, specifically at rounds 30 (early stage), 100 (middle stage), and 200 (late stage).

- Round 30: At the early stage, the importance scores between clients are relatively low, which is expected under heterogeneous data distributions. Each client’s personalized model primarily learns from its local data, resulting in significant divergence among models.
- Round 100: By the middle stage, as aggregation progresses, certain clients begin to show higher importance scores with others, indicating that the importance matrix facilitates convergence by selectively enhancing collaboration.
- Round 200: In the late stage, most clients exhibit high mutual importance scores, suggesting that their models have converged toward similar representations. Nonetheless, a few clients still maintain lower scores due to the distinctive characteristics of their local data.

In conclusion, the evolution of importance scores illustrates the effectiveness of the importance matrix in promoting collaboration among clients.

#### 4.2.5. Hierarchical Weight Fusion Analysis

**Figure 4:**
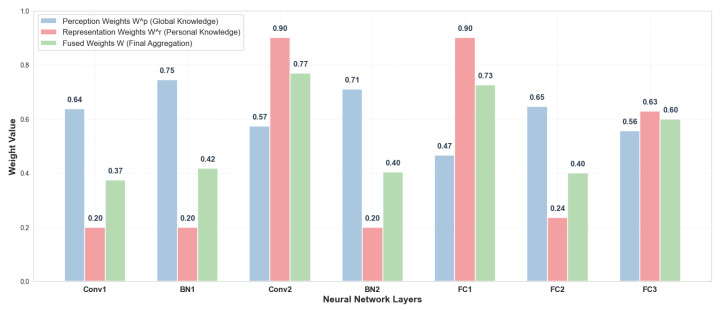
Hierarchical distributions of perceptual, representational, and fused weights generated by DHN on CIFAR-10 under a 5-way 5-shot setting.

To implicitly explore the impact of the adaptive fusion mechanism, we visualize the distributions of perception weights $W_{p}$, representation weights $W_{r}$, and the final fused weights *W* generated by the DHN. Figure 4 displays the weight values across the seven layers of the CNN model (Conv1, BN1, Conv2, BN2, FC1, FC2, FC3) for a representative client on CIFAR-10 under a 5-way 5-shot setting.

As observed in Figure 4, the fusion behavior exhibits a nuanced and biologically plausible adaptability. The Perception Branch ($W^{p}$, blue bars) acts as a stabilizer, maintaining consistent contributions across most layers to preserve shared generalized knowledge. In contrast, the Representation Branch ($W^{r}$, red bars) demonstrates selective activation. It explicitly dominates in layers critical for semantic abstraction, such as Conv2 (extracting complex patterns) and FC1 (transforming features to semantics), where peaks reach approximately 0.90. Conversely, in low-level statistical layers like BN1 and BN2, the Representation Branch remains suppressed. Unlike static aggregation, pFedH2A adjusts the reliance on global versus local knowledge based on the semantic role of each layer, thereby achieving a fine-grained balance essential for few-shot scenarios.

It is important to note that the fusion coefficients and branch weights remain within a stable range throughout the training. The explicit division of labor across layers confirms that the aggregation avoids the collapsing to a single branch or the saturation of weights, ensuring the effective utilization of the dual-branch design.

## 5. Conclusions

In this paper, we propose pFedH2A, a brain-inspired personalized federated learning framework for few-shot scenarios. Our method addresses two key challenges in federated few-shot learning: the lack of hierarchical modeling in existing aggregation strategies and the rigidity of conventional inductive biases under distributional shifts. Crucially, through a fine-grained hierarchical aggregation strategy, this study effectively addresses the trade-off between personalization and generalization. Our analysis reveals that the model prioritizes global generalization in shallow layers driven by perception weights, while shifting towards local personalization in deep layers driven by representation weights. This dynamic balancing acts as a flexible inductive bias, allowing the framework to generalize across clients without compromising local discriminative power. Validating this design, our extensive experiments demonstrate significant performance gains over existing pFL baselines. Specifically, under the 20-way five-shot setting on CIFAR-100, pFedH2A achieves an accuracy improvement of 3.72% under random heterogeneous allocation and 4.6% under cluster-sharing allocation compared to the second-best approach.

Despite promising results, pFedH2A has potential limitations that warrant further investigation. First, the maintenance of the client importance matrix involves pairwise comparisons. As the number of clients increases significantly, this may introduce computational overheads. Future work could explore sparse matrix techniques or clustering-based approximations to address this. Second, the sensitivity of the model to specific hyperparameters suggests that the integration of an adaptive optimization mechanism could further enhance robustness in dynamic federated environments. Third, this work currently lacks a formal proof of convergence due to the complex bilevel nature of the optimization. While our experiments demonstrate consistent empirical stability, establishing rigorous theoretical guarantees for such non-convex hypernetwork settings remains an open challenge reserved for future research.

## Figures and Tables

**Table 1:** Accuracy comparison of methods across various datasets and few-shot learning settings (denoted as (C,K), where *C* represents the number of classes and *K* represents the number of samples per class) under random heterogeneous allocation.

Method	MNIST				CIFAR-10				CIFAR-100			
	(3, 5)	(3, 20)	(5, 5)	(5, 20)	(3, 5)	(3, 20)	(5, 5)	(5, 20)	(5, 5)	(5, 20)	(20, 5)	(20, 20)
FedAvg	0.9077	0.9220	0.8799	0.9485	0.3121	0.4012	0.2854	0.3669	0.1002	0.1896	0.1077	0.1371
FedFomo	0.9069	0.9355	0.9114	0.9451	0.4157	0.5302	0.3602	0.4101	0.3572	0.4903	0.2101	0.3016
FedBN	0.9176	0.9548	0.8956	0.9399	0.3202	0.4223	0.2988	0.3851	0.3340	0.4851	0.1365	0.1845
pFedHN	0.9143	0.9401	0.8959	0.9406	0.4540	0.5724	0.3933	0.4863	0.2910	0.3896	0.1480	0.1853
FedFSL	0.9014	0.9746	0.9096	0.9601	0.5231	0.6278	0.4003	0.5428	0.2430	0.4269	0.1874	0.2879
pFedFSL	0.9234	0.9732	0.9326	0.9747	0.5757	0.7310	0.4557	0.5890	0.4832	0.6489	0.2345	0.3968
pFedH2A	**0.9494**	**0.9801**	**0.9401**	**0.9753**	**0.6656**	**0.7561**	**0.4755**	**0.6152**	**0.5362**	**0.6733**	**0.2717**	**0.4012**

**Table 2:** Accuracy comparison of methods across various datasets and few-shot learning settings (denoted as (C,K), where *C* represents the number of classes and *K* represents the number of samples per class) under cluster-sharing allocation.

Method	MNIST				CIFAR-10				CIFAR-100			
	(3, 5)	(3, 20)	(5, 5)	(5, 20)	(3, 5)	(3, 20)	(5, 5)	(5, 20)	(5, 5)	(5, 20)	(20, 5)	(20, 20)
FedAvg	0.9213	0.9495	0.8978	0.9433	0.3233	0.4085	0.3156	0.3656	0.2547	0.3946	0.1464	0.1745
FedFomo	0.9380	0.9708	0.9122	0.9619	0.5311	0.5922	0.4488	0.5313	0.4604	0.5670	0.2315	0.3224
FedBN	0.9326	0.9492	0.9273	0.9553	0.5044	0.5617	0.4127	0.5045	0.4304	0.5657	0.1845	0.2642
pFedHN	0.9367	0.9619	0.9240	0.9583	0.5881	0.6256	0.4767	0.5797	0.4480	0.5633	0.2107	0.2965
FedFSL	0.9130	0.9538	0.9190	0.9462	0.5547	0.7030	0.4593	0.6003	0.4458	0.5603	0.2809	0.3001
pFedFSL	0.9386	0.9740	0.9400	0.9741	0.6529	0.7388	0.5406	0.6681	0.5997	0.6746	0.3406	0.4870
pFedH2A	**0.9688**	**0.9892**	**0.9569**	**0.9780**	**0.7339**	**0.8393**	**0.6180**	**0.6974**	**0.6738**	**0.7523**	**0.3866**	**0.5121**

**Table 3:** Accuracy comparison of pFedH2A and its ablated methods across various datasets and few-shot learning settings under cluster-sharing allocation.

Method	MNIST		CIFAR-10		CIFAR-100	
	(5, 5)	(5, 20)	(5, 5)	(5, 20)	(5, 5)	(5, 20)
Variant 1	0.9318	0.9773	0.5231	0.6576	0.5722	0.7412
Variant 2	0.9280	0.9680	0.5345	0.6438	0.5671	0.7239
Variant 3	0.9025	0.9681	0.5313	0.6436	0.6028	0.7388
Variant 4	0.9400	0.9741	0.5406	0.6681	0.5997	0.6746
Variant 5	0.9504	0.9701	0.5820	0.6981	0.6314	0.6991
pFedH2A	0.9569	0.9780	0.6180	0.6974	0.6738	0.7523

**Table 4:** Accuracy comparison of pFedH2A under different numbers of clusters on 5-way 5-shot tasks, including maximum and average accuracy over five runs.

Allocation Method	Clusters	MNIST		CIFAR10		CIFAR100	
		Max	Avg	Max	Avg	Max	Avg
**Cluster Sharing**	1	0.9840	0.9697	0.6549	0.6270	0.8068	0.7434
	3	0.9614	0.9453	0.6326	0.6026	0.7280	0.6583
	5	0.9569	0.9498	0.6180	0.5758	0.6738	0.6191
	10	0.9391	0.9335	0.5825	0.5463	0.6179	0.5971
	15	0.9413	0.9288	0.5708	0.5293	0.5571	0.5407
	30	0.9400	0.9337	0.4773	0.4579	0.5400	0.5143
**Random Heterogeneous**	–	0.9401	0.9320	0.4755	0.4583	0.5362	0.5036

## Data Availability

Data are contained within the article. The source code of this work is publicly available at https://github.com/yf-miao/pFedH2A, accessed on 21 January 2026.
